# The Relationship Between Plasma Eosinophil Count and Coronary Artery Ectasia

**DOI:** 10.4021/cr280w

**Published:** 2013-10-15

**Authors:** Mehmet Demir, Serdar Keceoglu, Mehmet Melek

**Affiliations:** a Bursa Yuksek Ihtisas Education and Research Hospital Cardiology Department, Bursa, Turkey

**Keywords:** Coronary artery ectasia, Eosinophil, Mean platelet volume, Inflammation, Endotel dysfunction

## Abstract

**Background:**

The pathophysiology of coronary artery ectasia (CAE) has not been clearly identified, although multiple abnormalities including arteritis, endothelial dysfunction, and atherothrombosis have been reported. It is known that eosinophils play an important role in inflammation and thrombosis. Also vascular anomalies such as aneurysm have been noted in patients with hypereosinophilic syndromes. We aimed to compare the numbers of eosinophil counts of the patients CAE versus controls.

**Methods:**

This study included 50 CAE patients (20 male, mean age 60.26 ± 10.6 years) and 30 control person (10 male, mean age 57.86 ± 11.6 years). These participants were performed concurrent routine biochemical tests and neutrophil, lymphocyte, eosinophil count and mean platelet volume (MPV) on whole blood count. These parameters were compared between groups.

**Results:**

Baseline characteristics of the study groups were comparable. CAE patients had a higher MPV value, eosinophil, neutrophil lymphocyte ratio (NLR) than controls (8.5 ± 1 vs 76.2 ± 1.6 fl and 0.198 ± 0.14 vs 0.093 ± 0.058 and 3.0 ± 2.5vs 1.14 ± 0.9; P < 0.001, 0.002 and 0.028 respectively).

**Conclusion:**

As a result, our study revealed a relationship between eosinophil count, NLR and MPV in patients with CAE.

## Introduction

Coronary artery ectasia (CAE) has been characterized as a localized or diffuse non-obstructive lesion of the epicardial coronary arteries with a luminal dilation exceeding 1.5-fold the normal adjacent segment or vessel diameter [1]. The prevalence of CAE varies from 1.2% to 4.7% among patients undergoing coronary angiography [2-5].

The etiopathogenesis of this coronary enlargement is completely unknown. Although the exact mechanisms leading to CAE are not clear up to now, atherothrombosis and endothelial dysfunction, and vasculit have been suggested as possible responsible factors. CAE has also been reported in association with various conditions such as congenital coronary anomalies, connective tissue diseases, and vasculitis [6, 7].

It is known that eosinophils play an important role in endothelial dysfunction, inflammation and thrombosis [8, 9]. Also vascular anomalies such as aneurysm have been noted in patients with hypereosinophilic syndromes. In addition, there are also reports that hypereosinophilic syndromes may be associated with radial, coronary, hepatic and ulnar artery aneurysms [10, 11].

The powerful vasocactive and procoagulant effects of eosinophils made us hypothesize that there might be a correlation between eosinophil concentration and CAE. As far as we know, there is no study performed until today about the association of blood eosinophil concentration with CAE. In our study, we compared eosinophil counts, between CAE patients and control groups.

## Materials and Method

The study group included 50 patients (20 male, mean age 53.9 ± 11 years) with isolated CAE who had irregularities with ectatic coronaries without any stenotic lesions under visual assessment. The control group consisted of 30 age-and gender-matched subjects (10 male, mean age 49.16 ± 9.2 years) who proved to have normal coronary angiograms. The indication for coronary angiography was either the presence of typical angina or positive or equivocal results of noninvasive screening tests for myocardial ischemia in both of the groups.

Physical examination, medical history of patients, blood biochemistry and transthoracic echocardiographic examination were evaluated in both groups to exclude systemic diseases. Patients with obstructive coronary artery disease (who had coronary stenotic lesions of > 20%), chronic renal failure, chronic liver disorders, chronic lung disease, moderate or severe valvular disease, hypertension, diabetes mellitus, congenital heart disease, left ventricular systolic dysfunction on echocardiography (EF < 50%), anemia, pregnancy, obstructive sleep apnea, hematological disorders, known malignancy, thyroid dysfunction, hypercholesterolemia, electrolyte imbalance, and drug history included anti-gout agent, antiinflamuar agent (steroid or nonsteroid), antiaggregan or anticoagulant agents, antihistaminic and any medication that can potentially interfere with the measurement of eosinophil counts were excluded from the study. Also patients had a recent history of an acute infection or and high body temperature > 38 °C, an inflammatory or allergic disease are excluded from the study.

The patients having a systolic blood pressure ≥ 140 mmHg and/or a diastolic blood pressure ≥ 90 mmHg and those taking antihypertensive drugs were accepted to be hypertensive. Diabetes was defined as a fasting blood glucose level > 126 mg/dL or current use of a diet or medication to lower blood glucose. Current cigarette smoking was defined as use of > 10 cigarettes/day at the time of diagnosis.

### Coronary angiography

Coronary angiograms were performed with a femoral approach using the Judkins technique without the use of nitroglycerin, adenosine, or a calcium channel blocker. All patients in the study population underwent elective coronary artery angiography using Siemens Axiom Artis DFC (Siemens Medical Solutions, Erlangen, Germany) following appropriate patient preparation. Coronary angiograms were judged withregard to smooth appearance, luminal wall irregularities, epicardial local or diffuse caliber reduction, and stenosis. Coronary artery ectasia was defined as dilation of the coronary artery > 1.5-fold the diameter of the adjacent normal coronary vessels according to Falsetti and Carroll [12].

### Laboratory tests

Biochemical parameters were analyzed spectrophotometrically on ArchitectC16000 (Abbott. USA) autoanalyzer using enzymatic-colorimetric assay.

For whole blood count (eosinophil count, Hematochryt, Hemoglobine, MCV, MPV, leukocytes, neutrophil, lymphocyte and platelets), the blood samples were collected in tubes with EDTA and analyzed on CELL-DYN 3700 (Abbott. USA) device using impedance and optic scatter method.

### Statistical analysis

SPSS 16.0 statistical program (SPSS Inc., Chicago, IL, USA) was used for statistical study. All values are given as mean ± standard deviation. Mean values of continuous variables were compared between groups using the Student t test or Mann-Whitney U test, according to whether normally distributed or not, as tested by the Kolmogorov-Smirnov test. A P value of less than 0.05 was considered significant.

## Results

Evaluating basic clinical and demographic characteristics, there was no statistically significant difference between two groups in terms of age, gender distribution, body mass index, and smoking status and biochemical parameters (Table 1).

**Table 1:** Comparison of Basic Clinical and Biochemical Features of Patients and Controls

	Patients (n = 50)	Controls (n = 30)	P value
Age (years)	60.26 ± 10.6	57.86 ± 11.6	NS
Sex (n.%) males	20 (40%)	10 (33%)	NS
Body mass index (BMI) (kg/m^2^)	29.8 ± 5.4	28.5 ± 4.6	NS
Smoking	9 (18%)	6 (20%)	NS
Fasting glucose (mg/dL)	95.7 ± 9	97.6 ± 8.5	NS
Creatinin (mg/dL)	0.75 ± 0.1	0.72 ± 0.2	NS
Total cholesterol (mg/dL)	211 ± 45	181 ± 36	NS
Trigliserid (mg/dL)	162.5 ± 65	151.9 ± 41	NS
TSH (µIU/mL)	1.7 ± 0.6	1.6 ± 0.4	NS

TSH: thyroid-stimulating hormone; NS: nonsignificant.

Given blood count parameters, in the group of CAE patients blood eosinophil count, neutrophil lymphocyte ratio(NLR) and MPV value, were significantly higher in comparison with the control group. There was no statistically significant difference between two groups with regard to leukocyte count, platelet count, hemoglobin and hematochrit level (Table 2).

**Table 2:** Comparison of Whole Blood Count Features of Patients and Controls

	Group	N	Mean	Std. Deviation	Std. Error Mean	P value
eosinophil	patient	50	0.1980	0.14913	0.02109	0.002
	control	30	0.0933	0.05833	0.01065	
neutrophil	patient	50	5.8100	3.14942	0.44540	0.016
	control	30	3.5667	1.85869	0.33935	
lymphocyte	patient	50	2.2820	0.89458	0.12651	0.000
	control	30	4.5333	3.28731	0.60018	
NLR	patient	50	3.0062	2.56963	0.36340	0.028
	control	30	1.1454	0.91072	0.16627	
MPV (fL)	patient	50	8.5080	1.01274	0.14322	0.000
	control	30	6.2800	1.63167	0.29790	
Platelet (10^3^/µL)	patient	50	2.3236E2	77.06180	10.89818	0.644
	control	30	2.3477E2	60.28992	11.00738	
Leukocyte (10^3^/µL)	patient	50	9.1340	2.65435	0.37538	0.257
	control	30	8.4767	2.10708	0.38470	
Hemoglobin (g/dL)	patient	50	13.8160	1.95786	0.27688	0.010
	control	30	13.4967	1.15146	0.21023	
Hematocrit (%)	patient	50	40.6100	3.49701	0.49455	0.577
	control	30	40.7300	3.49000	0.63718	

Eosinophil count, MPV and MLR were found to be significant parameters using logistic regression analysis (Table 3).

**Table 3:** The Independent Relationship of MPV, Eosinophil and NLR

Model		Unstandardized Coefficients		Standardized Coefficients	t	Sig.	95% Confidence Interval for B	
		B	Std. Error	Beta			Lower Bound	Upper Bound
1	(Constant)	-0.828	0.197		-4.204	0.000	-1.221	-0.436
	MPV	0.189	0.025	0.650	7.547	0.000	0.139	0.239
2	(Constant)	-0.934	0.183		-5.113	0.000	-1.297	-0.570
	MPV	0.179	0.023	0.615	7.739	0.000	0.133	0.225
	eosinophil	1.157	0.291	0.316	3.973	0.000	0.577	1.737
3	(Constant)	-0.924	0.173		-5.351	0.000	-1.267	-0.580
	MPV	0.163	0.022	0.559	7.262	0.000	0.118	0.208
	eosinophil	1.113	0.276	0.303	4.036	0.000	0.564	1.662
	NLR	0.052	0.016	0.245	3.191	0.002	0.020	0.085

a. Dependent Variable: coronary artery ectasia.

## Discussion

In our study we have found significantly differences in eosinophil count, NLR and MPV between CAE patients and control group.

The pathophysiology of CAE has not been clearly identified yet, although multiple abnormalities including inflammation, endothelial dysfunction, vasculitis, and atherothrombosis have been reported [5]. CAE in association with connective tissue disorders such as scleroderma, in Ehlers-Danlos syndrome, also in syphilitic aortitis14and in Kawasaki disease [13].

Previous studies have demonstrated that CRP and NLR were higher in patients with CAE than in control participants. The increased levels of CRP and NLR may suggest that these markers may be used in clinical practice for the assessment of the inflammatory status of CAE [14-16].

As far as we know, there is no study available in the literature about the association between CAE and eosinophil count.

Eosinophils cause coagulation system activation and platelet activation, also cause inflammation and aneurysm. Additionally eosinophils play role vascular injury [10].

In our study we have found significantly differences in MPV and NLR between CAE patients and control group. Also, our findings are consistent with previous studies [14, 17]. Additionally when 2 groups were compared in our study, eosinophil count of the patients having CAE were significantly higher than control groups.

Eosinophils are equipped with several granule-associated molecules which play a role in the occurrence of thrombosis and vascular injury. Eosinophils generate an increased tendency to thrombosis through leukocyte, platelet stimulation and release of tissue factor [18, 19]. All these effects contribute to procoagulation through preventing the activation of thrombin and endorsing fibrin formation. Eosinophils store and release tissue factor as well as other cationic proteins. Major basic protein, eosinophilic cationic protein activates platelets and promotes thrombus formation by inhibiting thrombomodulin in hypereosinophilic syndromes and allergic diseases. Activated eosinophils and secreted eosinophil granule proteins were most evident within the necrotic and later stage thrombotic lesions and were found mainly within the areas of acute tissue damage in the endocardium and in the walls of small blood vessels. These findings suggest that eosinophil granule proteins are involved in vascular injury, also eosinophils may effect cardiovascular system through inflammatory cell infiltration [20, 21].

Recent studies showed that eosinophils were associated arterial tortuosity, dilatation and aneurysm inpatients with hipereosinophilic syndromes [22].Terai et al showed that relationship between coronary artery aneurysm and eosinophilia in patients with Kavasaki disease [23].

Major basic protein, eosinophilic cationic protein and eosinophil-derived neurotoxin are the primary mediators of eosinophil-associated toxicity to human tissue and may be eosinophilic myocarditis, pneumonitis, dermatitis, neuropathy and vasculitis [10].

Recently speculated that hypereosinophilic syndromes may be associated with radial, coronary, hepatic and ulnar artery aneurysms. Eosinophilic infiltrations have been implicated in the development of de novo coronary aneurysms [24].

Furthermore, eosinophilic vasculitis with medial necrosis has been identified at autopsy in otherwise healthy individuals with spontaneous coronary dissection or rupture. It has therefore been proposed that cytotoxic substances released from perivascular eosinophils may result in direct medial destruction, predisposing to aneurysmal formation or spontaneous intimal dissection and sudden cardiac death [25].

The powerful vasoactive, inflammatuar and procoagulant effects of eosinophils made us hypothesize that there might be a correlation between eosinophil concentration and CAE. In the literature, there is no study investigating the association between CAE and eosinophils. Our study is of importance with regard to this matter, we investigated the effect of eosinophil concentration on CAE patients.

The most important restriction of our study is the limited number of patients. Another limitation. Further studies are required to determine the relation between eosinophil count and CAE.

Our results may contribute to etiopathogenesis of CAE and pathophysiological mechanisms of increased prevalence of cardiovascular morbidity and mortality risk in these patients. Increased the concentration of eosinophil might be explained with vascular destruction, endotelial dysfunction and thrombosis in CAE patients.
